# P-531. Mitochondrial DNA Haplogroups and Circulating Immune Cell Phenotypes in Veterans with and without HIV

**DOI:** 10.1093/ofid/ofae631.730

**Published:** 2025-01-29

**Authors:** Aiwei Yan, Suman Kundu, David Samuels, Samuel S Bailin, Ke Xu, Kaku So-Armah, Adeel A Butt, Mariana Gerschenson, Matthew B Goetz, Russell Tracy, Vincent Marconi, Amy Justice, Matthew Freiberg, John R Koethe, Todd Hulgan

**Affiliations:** Vanderbilt University Medical Center and VA Tennessee Valley Healthcare System, Nashville, Tennessee; Vanderbilt University Medical Center and VA Tennessee Valley Healthcare System, Nashville, Tennessee; Vanderbilt University School of Medicine, Nashville, Tennessee; Vanderbilt University Medical Center, Nashville, Tennessee; Yale University School of Medicine, West Haven, Connecticut; Boston University School of Medicine, Boston, MA; Weill Cornell Medicine, Doha, Ad Dawhah, Qatar; University of Hawaii-Manoa, John A. Burns School of Medicine, Honolulu, Hawaii; VA Greater Los Angeles Healthcare System, Los Angeles, California; University of Vermont-Lerner College of Medicine, Burlington, Vermont; Emory University, Atlanta, Georgia; Yale School of Medicine, West Haven, CT; Vanderbilt University Medical Center, Nashville, Tennessee; Vanderbilt University Medical Center, Nashville, Tennessee; Vanderbilt University Medical Center, Nashville, Tennessee

## Abstract

**Background:**

HIV infection and mitochondrial function both influence cellular immune responses, but little is known about this relationship. We examined associations between mitochondrial DNA (mtDNA) haplogroups and peripheral blood mononuclear cell (PBMC) phenotypes among people with HIV (PWH) and without HIV in the Veterans Aging Cohort Study Biomarker Cohort (VACS-BC).

**Methods:**

VACS-BC participants had mtDNA haplogroups determined from genome-wide genotyping and PBMC phenotyping by flow cytometry. Analyses included Kruskal-Wallis and Wilcoxon rank sum tests stratified by self-reported ancestry (non-Hispanic Black or White) and HIV status. Significant univariate associations were assessed in linear regression models adjusted for age and plasma viral load < 50 copies/mL among PWH. These exploratory analyses were not corrected for multiple comparisons.

**Results:**

1939 Veterans (1266 PWH; 95% male; median age 52 years [range 25-91]) had data available. In Black PWH (N=991), African haplogroup L3 was associated with lower % senescent (CD28-negative) CD4+ T cells (p=0.05), and haplogroup L2 was associated with higher % TEMRA CD8+ T-cells (p=0.03). In White PWH (N=275), European haplogroup T was associated with lower % CD38+ CD4+ T-cells (p=0.02) and haplogroup J with lower % IL4+ CD4+ T-cells (p=0.001). Haplogroups T and J were also associated with lower (p=0.05) and higher (p=0.02) % transitional memory CD8+ T-cells, respectively. Different age-adjusted associations were seen in Veterans without HIV, with the most prominent between haplogroups T and L1 and lower % total (CD14+) and classic (CD14+/CD16-) monocytes (p-value range=0.02-0.005).

**Conclusion:**

MtDNA haplogroups were associated with cellular phenotypes, and associations differed by HIV status. Notable findings among PWH were in senescent, CD38+, and IL4-producing CD4+, and effector memory CD8+ T-cells, all important components of immune responses to viral infection. Haplogroup associations with monocyte populations in persons without HIV are intriguing and deserve further study. Limitations include those inherent in PBMC flow cytometry, cross sectional design, and small sample sizes for some subgroups. Validation in independent cohorts and mechanistic studies are needed.

**Disclosures:**

**Adeel A. Butt, MD, MS**, Gilead Sciences: Grant/Research Support **John R. Koethe, MD**, Gilead Sciences: Advisor/Consultant|Gilead Sciences: Grant/Research Support|Merck & Co.: Advisor/Consultant|Merck & Co.: Grant/Research Support

